# Multifunctional Polymer Nanoparticles for Dual Drug Release and Cancer Cell Targeting

**DOI:** 10.3390/polym9060213

**Published:** 2017-06-09

**Authors:** Yu-Han Wen, Tsung-Ying Lee, Ping-Chuan Fu, Chun-Liang Lo, Yi-Ting Chiang

**Affiliations:** 1 Department of Biomedical Engineering, National Yang Ming University, Taipei 11221, Taiwan; qooaz0815@gmail.com (Y.-H.W.); ivan173962@gmail.com (T.-Y.L.); momo519076@hotmail.com (P.-C.F.); 2 Department of Pharmacy, China Medical University, Taichung 40402, Taiwan

**Keywords:** polymeric nanoparticles, pH-sensitive, multifunctional polymer, imiquimod

## Abstract

Multifunctional polymer nanoparticles have been developed for cancer treatment because they could be easily designed to target cancer cells and to enhance therapeutic efficacy according to cancer hallmarks. In this study, we synthesized a pH-sensitive polymer, poly(methacrylic acid-*co*-histidine/doxorubicin/biotin) (HBD) in which doxorubicin (DOX) was conjugated by a hydrazone bond to encapsulate an immunotherapy drug, imiquimod (IMQ), to form dual cancer-targeting and dual drug-loaded nanoparticles. At low pH, polymeric nanoparticles could disrupt and simultaneously release DOX and IMQ. Our experimental results show that the nanoparticles exhibited pH-dependent drug release behavior and had an ability to target cancer cells via biotin and protonated histidine.

## 1. Introduction

Nanoparticle-based drug delivery systems have been used for cancer therapy for several decades. Among these nanocarriers, polymeric nanoparticles showed more stable and easy preparation by various manufacturing methods [1]. Polymeric nanoparticles with good design could evade recognition by the reticuloendothelial system (RES) and prolong the circulation time in blood vessels [2], resulting in increasing tumor-targeting probability by enhanced permeability and retention (EPR) effects [2]. Polymeric nanoparticles conjugated with targeting ligands (such as biotin [3], folate [4], transferring [5], and glucose [6]) not only enhanced tumor accumulation, but also improved cancer internalization. Additionally, polymeric nanoparticles by chemical conjugation or physical encapsulation of low permeability and low solubility drugs inside the core could become a platform for drug delivery. Polymeric nanoparticles could release payloads triggered by unique characteristics of tumor tissues, such as pH, ROS, hypoxia, and enzymes to improve drug therapeutic index [7,8].

Imiquimod (IMQ), a synthetic toll-like receptor 7 (TLR-7) agonist is an FDA-approved immunotherapy drug which used in actinic keratosis, basal cell carcinoma, and Bowen’s disease treatment [9]. TLR-7 could sense viral single-stranded RNA, guanosine, and small molecule agonists, followed by activating dendritic cells (DCs) to elicit IL-12, IL-6, and IFN-α [10,11]. Although imiquimod (IMQ) is a potent vaccine adjuvant in cancer treatment, its poor water solubility limits systemic absorption of IMQ in the animal body. Yong et al. mixed water-soluble polymer poly(*g*-glutamic acid) and hydrophobic drugs (IMQ and paclitaxel) in DMSO to prepare micro-dispersion powders. This formulation was well-dispersed in water without aggregation and precipitation. Their study showed that a IMQ and paclitaxel combined polymer solution injected into the tumor site could dramatically inhibit tumor growth, as compared to IMQ and paclitaxel alone [12]. 

**Figure 1:**
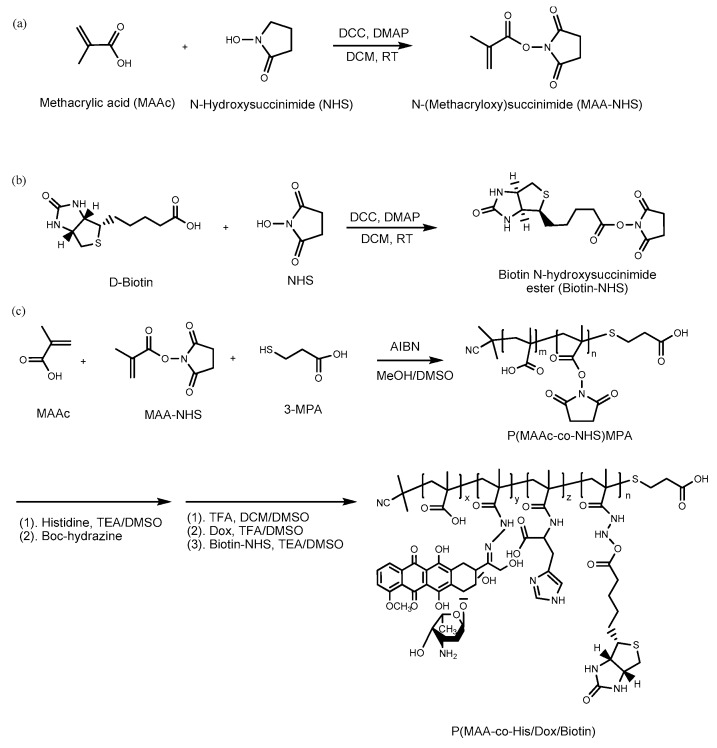
Synthesis protocol of (**a**) MAA–NHS, (**b**) biotin–NHS, and (**c**) P(MAAc-*co*-His/DOX/Biotin) copolymers.

pH-sensitive polymer nanaoparticles are one of the most important drug carriers for cancer therapy because they could respond to acidic pH in the tumor microenvironment (pH 6.5–7.2) [13] and cancer intracellular organelles, such as lysosomes and endosomes (pH 6.0–4.0) [14]. In this study, we synthesized a pH-sensitive copolymer, poly(methacrylic acid-*co*-histidine/biotin) (P(MAAc-*co*-His/Biotin)) to conjugate doxorubicin (DOX) via hydrazone linkers to form P(MAAc-*co*-His/DOX/Biotin) (abbreviated as HBD) as shown in Figure 1. The copolymers could self-assemble and physically encapsulate IMQ to form nanoparticles by a dialysis method. After cell internalization, the hydrazone linkage was pH-sensitive and can be hydrolytically degraded to release conjugated DOX. Additionally, HBD nanoparticles could release IMQ in an accelerated manner due to the protonation of PMAAc and histidine at low pH (the p*K*a values of PMAAc and histidine were 5.5 and 6.5, respectively) [15]. The protonated histidine could also induce nanoparticle-cell interaction to improve accumulation of nanoparticles in cancer cells [16]. Otherwise, HBD nanoparticles could actively target cancer cells through binding the cancer-cell overexpressed biotin receptors [17]. In this study, the optimal composition of HBD nanoparticles was investigated. The drug release behaviors and in vitro targeting ability of nanoparticles were also analyzed.

## 2. Materials and Methods

### 2.1. Materials

Methacrylic acid (MAAc), 3-mercaptopropionic acid (3-MPA), and sodium bicarbonate (NaHCO_3_) were purchased from Acros (Morris Plains, NJ, USA). d-biotin was purchased from Carbosynth (Berkshire, UK). 2,2′-Azobisisobutyronitrile (AIBN) was purchased from UniRegion Bio-Tech (Hsinchu, Taiwan). Histidine, *N*-hydroxysuccinimide (NHS), *N,N*′-Dicyclohexylcarbodiimide (DCC), *tert*-butyl carbazate and trifluoroacetic acid (TFA) were purchased from Sigma-Aldrich (St Louis, MO, USA). Doxorubicin (DOX) was purchased from LC Laboratories (Woburn, MA, USA). 4-Dimethylaminopyridine (DMAP) was purchased from Alfa Aesar (Heysham, Lancashire, UK). Dichloromethane (DCM), dimethyl formamide (DMF), dimethyl sulfoxide (DMSO) and isopropyl alcohol (IPA) were obtained from ECHO (Miaoli, Taiwan). AIBN was recrystallization from MeOH before use. DCM, DMF, and DMSO were dried and distilled with calcium hydride before use.

### 2.2. Synthesis of Methacrylic Acid N-hydroxysuccinimide Ester (MAA–NHS)

MAAc (0.4 mL, 4.69 mmol), NHS (0.8 g, 6.95 mmol), DCC (1.45 g, 7.02 mmol), and DMAP (0.17 g, 1.39 mmol) were dissolved in DCM (10 mL) in a two-neck round bottom under nitrogen. The reaction was conducted at 4 °C for 24 h. Acetic acid (0.4 mL, 6.99 mmol) was then added and reacted for 30 min. The reaction mixture was filtered to remove 1,3-dicyclohexyl urea (DCU), extracted progressively twice with 8% NaHCO_3_ and three times with water, and evaporated by a rotary evaporator to obtain the pure product. 

### 2.3. Synthesis of Biotin N-hydroxysuccinimide Ester (Biotin–NHS)

Biotin (0.5 g, 2.05 mmol), NHS (0.354 g, 3.08 mmol), DCC (0.64 g, 3.1 mmol), and DMAP (0.125 g, 1.03 mmol) were dissolved in anhydrous DMF (10 mL) for a 24 h reaction at 4 °C. After reaction, acetic acid was added and the DCU precipitation was removed by filtration. The crude product was precipitated by cold ether [18]. The final product was obtained from recrystallization twice in IPA. 

### 2.4. Synthesis of BD and HBD Copolymers

First, AIBN (7.6 mg, 0.046 mmol), MAAc (54 mg, 0.621 mmol), MAA–NHS (0.3 g, 1.638 mmol), and 3-MPA (122 mg, 1.15 mmol) were dissolved in MeOH (1.5 mL)/DMSO (1.5 mL) co-solvent. The reaction was performed at 70 °C for 24 h and then precipitated to obtain P(MAAc-*co*-NHS) copolymers. Second, P(MAAc-*co*-NHS) and histidine was dissolved in DMSO/TEA (97:3) and stirred at 70 °C for 72 h. After 72 h, the excess amounts of boc-hydrazine was added and reacted at 37 °C for 24 h. This solution was added into cold ether to obtain a sticky and yellow copolymer. Subsequently, the boc-protecting group of the copolymer was removed by mixing with DCM (6 mL), MeOH (2 mL), and TFA (6 mL) at 37 °C for 2 h. The organic solvent and remained TFA were removed by a rotary evaporator to obtain P(MAAc-*co*-His/Hydrazine) copolymers. Finally, to conjugate DOX on copolymers via hydrazone linkers, the copolymers were dissolved in DMSO containing 0.5% TFA and added DOX to the solution at 37 °C with stirring. After 24 h, biotin–NHS and TEA were added to the solution and reacted overnight. To remove free DOX and biotin–NHS, the mixture was dialyzed against DMSO by dialysis bag (MWCO 1K) for 48 h and then purified by a Sephadex™ LH-20 column. The resulting solution was froze and lyophilized to obtain HBD copolymers. P(MAAc-*co*-biotin/DOX) (abbreviated as BD) copolymers were prepared under the same protocol without adding histidine in the second step. The polymer composition was calculated by ^1^H NMR in d6-DMSO. The molecular weight (*M*_w_) and polydispersity index (PDI) was determined by gel permeation chromatography (GPC).

### 2.5. Preparation of IMQ-Loaded Nanoparticles

Five milligrams of copolymers and fixed amount of IMQ were dissolved in DMSO (5 mL). The solution was introduced into dialysis bag (MECO 6–8K, Spectrum Labs, Rancho Dominguez, CA, USA) and dialyzed against purified water for 24 h. After that, the precipitates were removed by centrifugation at 3000 rpm for 20 min. The particle size and polydispersity index (PDI) were determined by dynamic light scattering (DLS) (Particulate system nano Plus, Norcross, GA, USA). The morphology of polymeric nanoparticles was observed by transmission electron microscopy (TEM; JEM-2000EX II, Tokyo, Japan) at accelerating voltage of 120 kV. The nanoparticles were dropped on copper grid coating with carbon, and then stained with 1% of phosphotungstic acid (PTA) for negative stain.

### 2.6. Measurement of Drug Release in Various pH Conditions

Nanoparticles were suspended in different pH aqueous solutions (pH 7.4, 6.5, 5.0 and 4.0) at 37 °C and were shaken at 40 rpm. The released drugs from nanoparticles were isolated by dialysis bag (MWCO 6–8K). At different time interval, the isolated solution was analyzed using HPLC system. Inspire C18™ column (5 μm, 250 mm × 4.6 mm) was used to separate the analytes. The wavelength for determining IMQ concentration by Shimadzu SPD 10A UV–VIS detector (Kyoto, Japan) as observed at 244 nm. The Shimadzu RF-10 AXL fluorescence detector (Kyoto, Japan) was used to measure DOX concentration at λ_ex_ = 480 nm/λ_em_ = 570 nm. The mobile phase was 70% CH_3_COONa (pH = 5.5) and 30% ACN at a flow rate of 0.5 mL/min.

### 2.7. Cellular Uptake Analysis by Flow Cytometry

4T1 mouse breast cancer cells were cultured in DMEM/F12 medium containing 10% fetal bovine serum (FBS), sodium pyruvate, non-essential amino acid (NEAA), and penicillin/ streptomycin at 37 °C. Cells (5 × 10^5^ cells/well) were then seeded in a six-well plate to evaluate the internalization of nanoparticles. After 12 h incubation, the cells were pre-treated with 400 μM of biotin for 1 h, and were then incubated with nanoparticles for 1 h. After incubation, the cells were suspended in cold PBS and were analyzed by BD FACSCalibur flow cytometer (San Jose, CA, USA). The fluorescent intensity of DOX in nanoparticles was monitored by the FL2 channel (San Jose, CA, USA).

## 3. Results and Discussion

### 3.1. Characterizations of BD and HBD Copolymers

**Figure 2:**
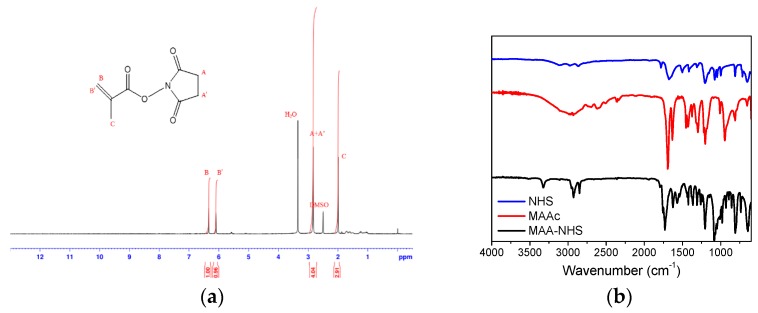
(**a**) ^1^H NMR and (**b**) FT-IR spectra of MAA–NHS.

**Figure 3:**
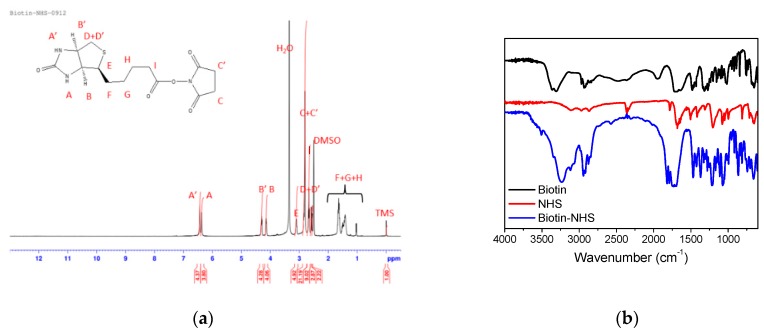
(**a**) ^1^H NMR and (**b**) FT-IR spectra of Biotin–NHS.

**Figure 4:**
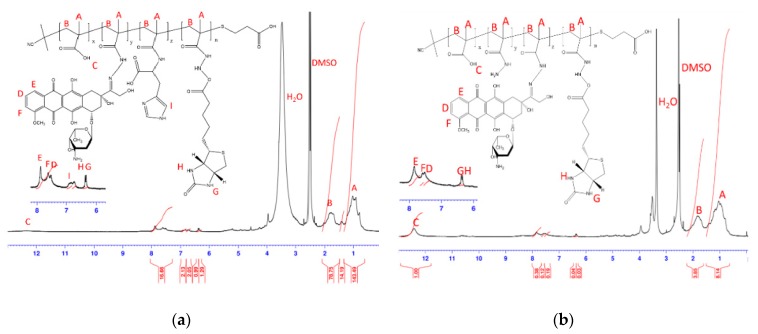
^1^H NMR spectrum of (**a**) HBD and (**b**) BD copolymers.

Before polymer preparation, MAA–NHS monomers and Biotin–NHS molecules were first synthesized via Steglich esterification. Figure 2 shows the ^1^H NMR and FT-IR spectrum of MAA–NHS. The chemical shifts of vinyl groups from MAA at 6.09 and 6.34 ppm (peak B’ and B) and methylene protons from NHS at 2.83 ppm (peak C) were observed. Additionally, except for the carboxyl group (2750–3250 cm^−1^ from MAAc) and hydroxyl group (3250 cm^−1^ from NHS), the vibrational frequencies for MAA–NHS were similar to those for MAAc and NHS. These experimental results all indicate that MAA–NHS was successfully prepared. Figure 3 shows the ^1^H NMR and FT-IR spectrum of Biotin–NHS. All experimental chemical shifts could assign to corresponding hydrogen atoms of Biotin–NHS; 6.4 and 6.5 ppm for –NH (from biotin), 4.1 and 4.3 ppm for –CH (from biotin), and 2.8 ppm for –CH2 (from NHS). FT-IR showed that Biotin–NHS showed two remarkable stretching vibrational peaks at 1660 cm^−1^ (N–O bond) and 1700 cm^−1^ (C=O bond), indicating that Biotin–NHS molecules were also successfully obtained. After small molecule preparation, the P(MAAc-*co*-NHS) copolymers were synthesized by free radical polymerization, using AIBN and 3-MPA as an initiator and a chain transfer reagent, respectively. According to the ^1^H NMR spectrum of P(MAAc-*co*-NHS), the molar ratio of NHS was about 40% (data not shown). By GPC determination, the molecular weight and PDI of P(MAAc-*co*-NHS) were 1370 and 1.56, respectively. P(MAAc-*co*-NHS) copolymers were then conjugated with biotin, DOX, or histidine to form BD and HBD copolymers. Figure 4A,B demonstrate the specific protons for BD and HBD copolymers, respectively. The methyl (0.8–1.3 ppm, peak A), methylene (1.5~2.2 ppm, peak B), and carboxyl signals (12.5 ppm, peak C) from PMAAc, amine signals (6.4 and 6.5 ppm, peak H and G) from biotin, and benzene groups (7.5–8.0 ppm, peak D, E and F) from DOX were observed in both BD and HBD copolymers. Additionally, the ^1^H NMR for HBD copolymers also contained the chemical shift of the imidazone ring at 6.5–7.0 ppm (peak I), indicating that histidine was successfully conjugated on HBD copolymers. The characterization of BD and HBD copolymers are shown in Table 1. The composition of DOX and biotin for both BD and HBD copolymers were similar; DOX and biotin were around 12–14 mol % and 3 mol % in copolymers, respectively. The composition of histidine in HBD copolymers was about 5 mol %. The amount of biotin molecules in copolymers were lower than expected, perhaps because the steric hindrance of DOX affected the conjugation efficiency.

### 3.2. Characterizations of BD and HBD Nanoparticles

**Figure 5:**
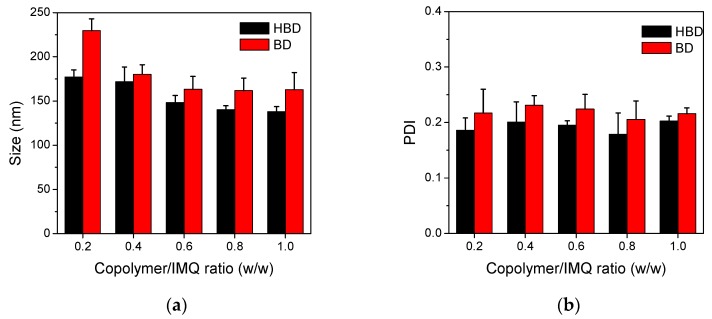
(**a**) Particle size and (**b**) PDI for of BD and HBD nanoparticles prepared from various copolymer/IMQ feed ratios.

To obtain the optimal size and size distribution of nanoparticles, copolymers and IMQ with various copolymer/IMQ feed ratios (from 0.2 to 1) were dissolved in DMSO and self-assembled to form nanoparticles by a dialysis method. Figure 5 illustrates the particle size and distribution of nanoparticles. The experimental results show that the particle sizes of HBD nanoparticles decreased from 177 nm to 138 nm as the feed ratio of IMQ increased. A similar decreasing tendency was observed in BD; the particle sizes decreased from 230 nm to 163 nm. Otherwise, all BD and HBD nanoparticles had similar PDI values closed to 0.2, indicating narrow size distributions of BD and HBD nanoparticles.

To understand the influence of copolymer/IMQ feed ratio on drug loading and surface charges, nanoparticles with the feed ratios of 0.2 and 1 were collected and analyzed. As shown in Table 2, the zeta potentials for all polymeric nanoparticles were about −27 mV, perhaps because almost 60 mol % of PMAAc in BD and HBD copolymers constructed the nanoparticles and contributed negative charges to the surface of nanoparticles. Otherwise, except for HBD nanoparticles with a copolymer/IMQ feed ratio of 0.2, the loading efficiencies for other nanoparticles were around 1.3 *w*/*w* %. Converting the E.E. values, it is expected that a higher copolymer/IMQ feed ratio contributed to lower drug content and smaller particle sizes. It is known that the characteristics of nanoparticles could directly affect cancer therapeutic efficacy. Particle sizes ranging from 10 to 200 nm could extravasate from the disorganized tumor vasculature to the tumor microenvironment due to tumor angiogenesis. A particle sizes were larger than 300 nm, nanoparticles could be easily recognized by reticuloendothelial system (RES) and decreased the circulation time in blood vessel [19]. Otherwise, anionic nanoparticles could repel BSA with negative charges, leading to reduced protein absorption and prolonged circulation time [20]. Therefore, our nanoparticles, around 200 nm in size and with negative surface charges, are considered to prevent large protein adsorption and accumulation in tumor sites.

**Figure 6:**
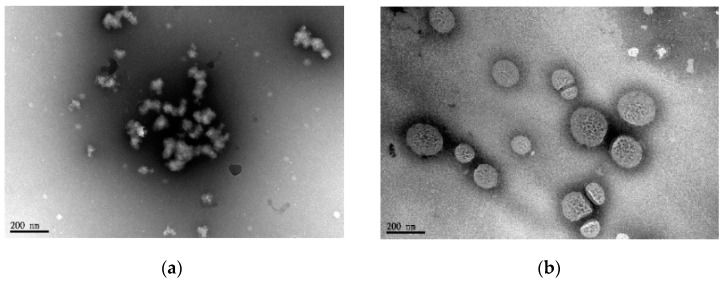
TEM images of (**a**) BD5 and (**b**) HBD5 polymeric nanoparticles.

TEM was used to directly observe the structures of nanoparticles. Figure 6 illustrates the morphology and particle size of BD and HBD nanoparticles stained negatively with PTA (1 *w*/*v* %). The TEM images suggested that BD nanoparticles were irregularly shaped and were constructed from small copolymer clusters. In contrast, the morphology of HBD nanoparticles was a porous nanosphere with around 150 nm in size. Although the E.E. values for both nanoparticles were similar, it could be reasonable to assume that HBD nanoparticles had a better IMQ/copolymer-packed structure than BD nanoparticles.

### 3.3. In Vitro Drug Release Behaviors of BD and HBD Nanoparticles

**Figure 7:**
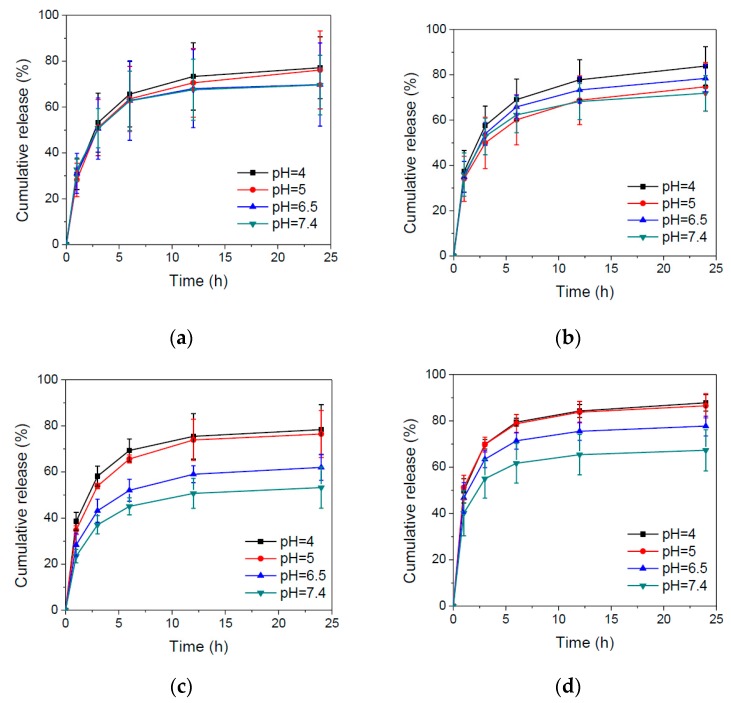
IMQ release profiles of (**a**) BD1, (**b**) BD5, (**c**) HBD1, and (**d**) HBD5 polymeric nanoparticles in various pH solutions.

pH-sensitive nanoparticles could release internal payloads in the tumor microenvironment or endosome/lysosome, thereby reducing side effects and enhancing therapeutic efficacy. The ideal condition is that drug-releasing profiles from nanoparticles exhibited significant differences in the neutral and acidic surroundings. Thus, IMQ release behaviors from nanoparticles were evaluated to understand which copolymer/IMQ feed ratio of nanoparticles met the pH-modulated release requirement. Figure 7 shows the IMQ release behaviors from different copolymer/IMQ feed ratio-manufactured nanoparticles at different pH. The experimental results demonstrate that BD nanoparticles did not show pH-triggered IMQ release behavior, while HBD nanoparticles revealed pH-dependent release of IMQ due to protonation of imidazole ring of histidine. Additionally, there is no significant difference of IMQ release in high copolymer/IMQ HBD nanoparticles. In contrast, low copolymer/IMQ HBD nanoparticles illustrated better pH-dependent release behavior. The reason is perhaps because a low copolymer/IMQ feed ratio of HBD nanoparticles had a denser structure. IMQ are able to induce squamous cell carcinoma (SCC) cell apoptosis via the intrinsic pathway at 50 μg/mL [21]. On the other hand, the low concentration of IMQ could also activate dendritic cells for cancer immunotherapy [22]. Our study indicated that HBD nanoparticles could release IMQ at both pH 6.5 (tumor microenvironment) and pH 5.0 (cancer intracellular endosome), which had the ability to activate the innate immune response by DCs and cause cancer cell apoptosis.

**Figure 8:**
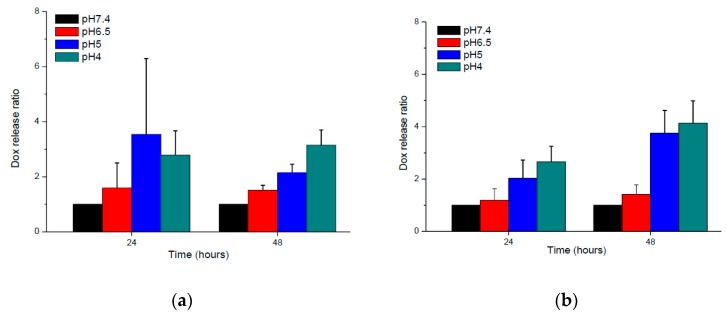
DOX release ratio of (**a**) BD5 and (**b**) HBD5 polymeric nanoparticles at different pH.

In cancer treatment, many acid-degradable linkers could be use to conjugate drugs and polymers for pH-triggered drug release. These specific linkers included acetal/ketal [23], benzoic imine [24], and hydrazone [25]. In this study, the hydrazone linkage was adopted because it is more stable as compared to acetal/ketal and benzoic imine in weak acid surroundings (pH 6.0–7.0). Figure 8 demonstrates the DOX release behaviors from BD and BDH nanoparticles at different pH. The experimental results show that both BD and HBD nanoparticles revealed pH-dependent DOX release behavior. The release rate of DOX from both nanoparticles increased as pH decreasing. At pH 4.0, the accumulated release of DOX from HBD nanoparticles after 48 h was approximately 1.3-fold that of the BD nanoparticles. The possible reason for the faster release of DOX from HBD nanoparticles is the increase of the hydrazone-hydrolysis probability contributed by the histidine protonation and porous nano-structure.

### 3.4. In Vitro Cellular Uptake

**Figure 9:**
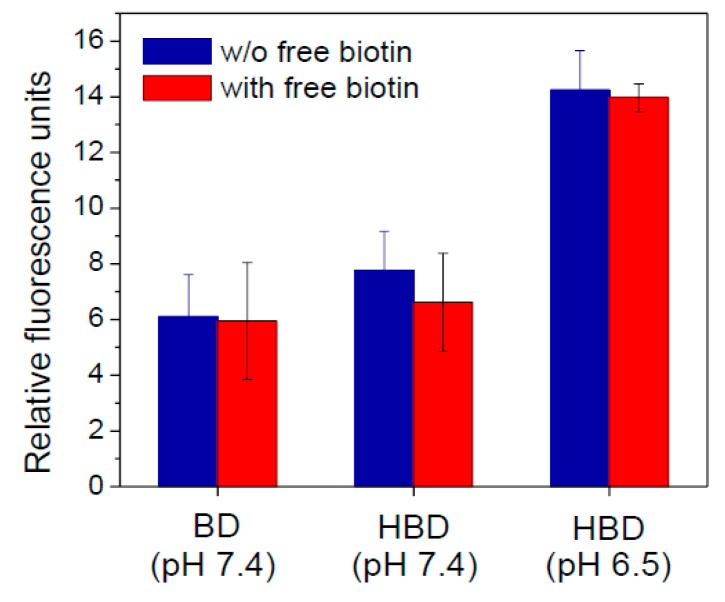
Cell uptake and competition test for BD5 and HBD5 nanoparticles in 4T1 cells.

In this study, biotin molecules outside the nanoparticles could trigger nanoparticles to recognize cancer cells by active targeting. Additionally, histidine molecules could be protonated and exhibit positive charges to induce adsorptive-mediated endocytosis. To evaluate the targeting ability of nanoparticles, 4T1 cells that which overexpressed biotin receptors were incubated with nanoparticles at pH 7.4 and 6.5 to confirm particle accumulation [3]. Additionally, the specific biotin-cell interaction was evaluated by a competition assay. Figure 9 shows the effects of the targeting abilities of nanoparticles on receptor-mediated endocytosis and adsorptive-mediated endocytosis. As compared to BD nanoparticles, HBD nanoparticles revealed higher cell accumulation at pH 7.4, indicating that nanoparticles could bind with 4T1 cells to increase cell uptake. Particularly, HBD nanoparticles showed the highest cell binding at pH 6.5, demonstrating that HBD nanoparticles targeted cancer cells not only by receptor-mediated endocytosis, but also by adsorptive-mediated endocytosis. Otherwise, after adding an excess of free biotin for the competition test, the accumulation of HBD nanoparticles at pH 7.4 was reduced and was similar to that of BD nanoparticles. In contrast, HBD nanoparticles at pH 6.5 still exhibited high cellular accumulation. Although cell targeting of HBD nanoparticles was inhibited by free biotin at pH 7.4, the loss of cell accumulation could be recovered by adsorptive-mediated endocytosis from the protonation of histidine at pH 6.5. These experimental results suggest that HBD nanoparticles exhibited dual targeting abilities that could enhance cell uptake and perhaps increase the therapeutic efficacy.

## 4. Conclusions

This study successfully prepared a cancer targeting and pH-sensitive nanoparticle to encapsulate DOX and IMQ. The experimental results suggest that the particle size of nanoparticles could be adjusted by the feed ratio of hydrophobic drug. Additionally, nanoparticles revealed pH-dependent drug release behaviors for both DOX and IMQ, and showed cancer-targeting ability in a simulated tumor microenvironment. The development of new small molecule drugs and protein drugs are high in risk and cost. A new approach is to encapsulate “old drugs” by multifunctional polymer nanoparticles. Drugs could be loaded into multifunctional polymer nanoparticles via chemical conjugation or physical encapsulation. Manipulation and functionalization of polymers enabled nanoparticles to control drug release, decrease side effects, improve stability and solubility of drugs and prolong the circulation time [26]. Multifunctional polymer nanoparticles could be easily prepared according to the disease requirements. Therefore, the use of well-designed multifunctional polymer nanoparticles could be an opportunity to overcome the dilemma of cancer treatment.

## Figures and Tables

**Table 1:** Composition of BD and HBD copolymers.

Code	In Feed (mol %)			In Copolymer (mol %) ^a^			*M*_w_^b^	PDI ^b^
	H	D	B	H	D	B		
BD	0	16%	8%	0	11.9%	3.6%	1510	3.53
HBD	16%	16%	8%	5.3%	14.1%	2.9%	850	1.55

^a^ The molar percentages of the histidine (H), DOX (D), and biotin (B) was calculated by ^1^H NMR; ^b^ The average molecular weight (*M*_w_) and polydispersity index (PDI) of copolymers were measured by GPC.

**Table 2:** Characteristic of HBD and BD polymeric nanoparticles with high and low copolymer/IMQ feed ratio.

Group	Copolymer (mg)	IMQ (mg)	Size (nm)	PDI	Zeta (mv)	E.E. ^a^ (%)
BD1	1	5	230 ± 12	0.22 ± 0.04	−27.9 ± 2.0	1.23 ± 212
BD5	5	5	163 ± 19	0.22 ± 0.01	−28.1 ± 2.4	1.29 ± 239
HBD1	1	5	177 ± 8	0.19 ± 0.02	−27.2 ± 2.3	0.66 ± 633
HBD5	5	5	138 ± 6	0.20 ± 0.01	−27.2 ± 27.	1.40 ± 466

^a^ E.E.: Encapsulation efficiency = (weight of IMQ in nanoparticles/weight of IMQ in feed) × 100%.
